# Microbial and Biochemical Profile of Different Types of Greek Table Olives

**DOI:** 10.3390/foods12071527

**Published:** 2023-04-04

**Authors:** Niki Mougiou, Antiopi Tsoureki, Spyros Didos, Ioanna Bouzouka, Sofia Michailidou, Anagnostis Argiriou

**Affiliations:** 1 Institute of Applied Biosciences, Centre for Research and Technology Hellas, Thermi, 57001 Thessaloniki, Greece; nmougiou@certh.gr (N.M.); adatsoureki@certh.gr (A.T.); sdidos@certh.gr (S.D.); ibouzouka@certh.gr (I.B.); sofia_micha28@certh.gr (S.M.); 2 Department of Food Science and Nutrition, University of the Aegean, Myrina, 81400 Lemnos, Greece; 3 Department of Medicine, Aristotle University of Thessaloniki, 54154 Thessaloniki, Greece

**Keywords:** microbiome, table olives, 16S rRNA, 18S rRNA, oleuropein, oleocanthal

## Abstract

Analysis of table olives microbiome using next-generation sequencing has enriched the available information about the microbial community composition of this popular fermented food. In this study, 16S and 18S rRNA sequencing was performed on table olives of five Greek popular cultivars, Halkidikis, Thassou, Kalamon, Amfissis, and Konservolia, fermented either by Greek style (in brine or salt-drying) or by Spanish style, in order to evaluate their microbial communities. Moreover, analytical methods were used to evaluate their biochemical properties. The prevailing bacterial species of all olives belonged to *Lactobacillaceae*, *Leuconostocaceae*, and *Erwiniaceae* families, while the most abundant yeasts were of the *Pichiaceae* family. Principal coordinates analysis showed a clustering of samples cured by salt-drying and of samples stored in brine, regardless of their cultivar. The biochemical evaluation of total phenol content, antioxidant activity, hydroxytyrosol, oleuropein, oleocanthal, and oleacein showed that salt-dried olives had low amounts of hydroxytyrosol, while Spanish-style green olives had the highest amounts of oleocanthal. All the other values exhibited various patterns, implying that more than one factor affects the biochemical identity of the final product. The protocols applied in this study can provide useful insights for the final product, both for the producers and the consumers.

## 1. Introduction

The olive tree, *Olea europaea* L., is a widely cultivated crop of major economic importance, known for the high nutritional value of its fruits [1]. Table olives are one of the most popular fermented foods of plant origin, with a world consumption of ca. 3 million tons in the year 2020 [2]. Olive fruits cannot be eaten directly from the tree due to their bitter taste, which is attributed mainly to the phenolic compound oleuropein. Thus, several procedures to remove bitterness were developed, with the three main trade preparations being the Greek or natural style, treated or Spanish style, and California style. In Greek style, olives are placed directly in brine for 8–12 months or in coarse salt (Throuba style), where osmosis along with microorganisms, mainly lactic acid bacteria (LAB) and yeasts, extract or enzymatically degrade oleuropein [3]. In Spanish style, alkaline treatment is used to remove bitterness and then olives are placed in brine, while in California style, the olives are first kept in brine, then subjected to lye treatment and then darkened by oxidation [4]. A new trend in table olives preparation is the use of starter cultures with probiotic properties that reduce the spoilage risk and result in a final product with improved organoleptic properties [5,6].

In the three preparation styles where a starter culture is not used, fermentation starts naturally by the autochthonous microbiota present on the olive fruit, as well as by the microbes existing in the processing environment [7]. The different microbial populations involved in the process depend on various intrinsic and extrinsic factors, such as salt concentration and pH of the brine, temperature, storage conditions, and more [6]. The majority of microorganisms involved in fermentation belong to LAB and yeasts, while in less amounts may be found bacteria belonging to *Enterobacteriaceae*, *Clostridium*, *Pseudomonas*, *Staphylococcus*, and occasionally molds [8]. LAB, specifically *Lactiplantibacillus plantarum* and *Lactiplantibacillus pentosus*, have strong β-glucosidase and esterase activities which are the enzymes that degrade oleuropein, thus causing debittering of the fruit [7]. Moreover, they produce lactic acid that causes a drop in the brine pH, preventing the growth of spoilage microorganisms and pathogens [7]. Bacteria from the *Enterobacteriaceae* family are commonly found at the first stages of fermentation when the pH of the brine is elevated [9,10]. If the brine pH stays high, *Enterobacteriaceae* will persist and their presence is associated with the formation of gas-pockets that soften the final product, lowering its quality [11]. Thus, quick acidification of the brine by LAB is desirable in order to inhibit the growth of *Enterobacteriaceae* [12].

Yeasts, such as *Wickerhamomyces anomalus*, *Saccharomyces cerevisiae*, *Pichia kluyveri*, and *Pichia membranifaciens*, are mainly found in brine and contribute to the taste and aroma of the final product, producing desirable metabolites and volatile compounds [13]. Moreover, yeasts enhance the growth of LAB [14] and degrade phenolic compounds [15]. On the other hand, the CO_2_ produced from yeasts may cause softening of the olives and spoilage of the end product at the early stages of fermentation [16]. Microorganisms that may be found in the final product and could be indicators of food spoilage and potential hazard are *Enterobacteriaceae* or *Escherichia coli*, *Staphylococcus aureus*, *Salmonella*, and the spore-forming bacteria of the genera *Bacillus* and/or *Clostridium* [17].

Greece devotes 80% of its cultivated land to olive growing [18] and is the second country in table olives production in the European Union after Spain, producing about 230 thousand tons in the year 2020 [19]. There are more than 50 Greek olive cultivars [18], but only half of the varieties are widely cultivated and used for olive oil and table olives production.

Halkidikis cultivar is economically the most important Greek cultivar for table olives, producing 90,000 tons of final product [20]. The olives from Halkidikis cultivar, that have a PDO certificate, are collected in two different time points and treated in different ways, resulting in two products: green Halkidikis olives, where mature but still green fruits are collected and treated in Spanish-style, and salt-dried black olives that are collected at the end of the growing season, when the fruit has turned black, and are placed directly in coarse salt, resulting in Throuba-style, meaning dehydrated, wrinkled olives. These olives are popular under the name Throuba Halkidikis. Another Greek olive cultivar, mainly treated by the salt-drying method, is Thassou or Thasitiki, where the final product, commonly named Throuba, is PDO certified and occupies 5% of the Greek market [21]. Throuba olives are of special interest as the fruit is left on the tree until full ripeness, which causes natural debittering of the fruit on the tree [22]. The mechanism behind this is not elucidated; a study in table olives made in 1932 suggested that the fruit gets colonized by the fungus *Phoma oleae* that causes degradation of oleuropein [23,24]; however, no more recent research has supported that. It is known that oleuropein degrades naturally as the olive fruit matures, due to decompartmentalization of the cells and subsequent contact of oleuropein with the enzyme β-glucosidase [25]. This debittering process is beneficial to the tree in order to make the fruit edible by animals, promoting seed dispersal. After collection of the fully mature fruits, the olives are placed directly in coarse salt, resulting in dehydration rather than fermentation, in a process called curing [17]. The cv. Amfissis or Konservolia Amfissis (PDO) is the second most widely used Greek cultivar for table olives, while cv. Kalamon (PDO) is the third, producing 65,000 and 50,000 tons of table olives, respectively [20]. Konservolia cultivar includes olives from many areas of Greece, such as Amfissis, Stilyda, Atalanti, and Rovies, which are all PDO certified and are treated either in Spanish or Greek style, resulting in different products.

For many years now, it has been acknowledged that culture-dependent techniques cannot fully reveal the richness of the microbial community of an ecosystem or food [26]. The advances in molecular biology, and more specifically, next-generation sequencing (NGS), have changed the way the olive microbiome is studied. Nowadays, microbial genomic DNA is isolated from olives and analyzed by NGS, in a culture-independent way, revealing a plethora of bacteria and yeast communities populating table olives [27]. In recent years, progress has been made in the analysis of Greek table olives’ microbiome from various cultivars [9,10,28,29,30,31,32].

The consumption of table olives and olive oil is linked to many health benefits, such as fewer incidents of chronic degenerative and cardiovascular diseases, as a result of the monounsaturated fatty acids they contain but mainly due to the high content of phenolic compounds [33,34,35,36]. The most abundant olive phenolic compounds are oleuropein, which is a secoiridoid responsible for the bitter taste of the fruit, and the simple phenols tyrosol and hydroxytyrosol. The phenolic content of an olive fruit is highly dependent on the cultivar, the cultivation techniques, and the environmental conditions [37]. Oleuropein is the most abundant compound in the fresh olive fruit, but all table olive treatments aim to eliminate it in order to remove the bitter taste of the fruit; thus, in the final edible product, its concentration is very low, if not undetected [38]. The decarboxylated dialdehyde derivative of oleuropein, oleocanthal, responsible for the burning sensation on the back of the throat after olive oil consumption, constitutes 10% of olive polyphenols and has many biological activities [39]. Another compound produced from the hydrolysis of oleuropein by β-glucosidases during fresh fruit processing and oil production is oleacein. Oleacein is lipophilic and may survive the human stomach conditions, and thus is considered responsible for the health benefits of olives’ consumption [40].

In this study, the microbiome of 18 samples of table olives produced from 5 different Greek cultivars, Halkidikis, Throuba Thassou, Kalamon, Amfissis, and Konservolia, was studied to compare their microbial communities. Moreover, a biochemical analysis, including determination of antioxidant capacity, total phenolic content, and amounts of oleuropein, oleacein, oleocanthal, and hydroxytyrosol, was performed to gain a better understanding of the final product.

## 2. Materials and Methods

### 2.1. Sample Description

In total, 18 table olive samples, belonging to 5 cultivars, 1 of them in 2 different fermentation styles, were purchased either unpacked or packed from the local market (Table 1). Amfissis cultivar is also known as Konservolia Amfissis, but we used the name Amfissis to distinguish it from Konservolia samples for which the cultivation area was not mentioned on the packaging. When modified atmosphere (MA) packaging was used to store samples, gas composition of 70% Ν_2_/30% CO_2_ was applied.

### 2.2. Library Construction and Sequencing

Twenty (20) grams of each olive sample were placed in a sterile disposable plastic container in the presence of 5 mL ddH_2_O and homogenized to a pulp using a polytron homogenizer (Polytron PT-MR 6100, Kinematica AG, Littau, Switzerland). Microbial DNA was then extracted using ~400 μL of the homogenized pellet, with the ZymoBIOMICS DNA Miniprep Kit (Zymo Research, Irvine, CA, USA). Qubit 4 Fluorometer was used to measure microbial DNA concentration using the Qubit^®^ dsDNA BR assay kit (Invitrogen, Carlsbad, CA, USA). Amplicon metabarcoding libraries were constructed as described in Michailidou et al., 2021 [41]. Briefly, the bacterial and fungal community of each sample was assessed by sequencing the V3–V4 hypervariable regions of the 16S rRNA gene and the V7–V8 hypervariable regions of the 18S rRNA gene, respectively. For 16S rRNA gene analysis, primers D-Bact-0341-b-S-17 and D-Bact-0008-a-S-16 were selected from Klindworth et al., 2013 [42], whereas for the amplification of 18S rRNA gene, primers FR1 and FF390 were selected from Prévost-Bouré et al., 2011 [43]. All libraries were constructed using the standard protocol provided by Illumina for 16S rRNA analysis (Part # 15044223 Rev. B). Paired-end sequencing was performed on a MiSeq platform, using the MiSeq^®^ reagent kit v3 (2 × 300 cycles) (Illumina, San Diego, CA, USA).

### 2.3. Bioinformatics and Data Analysis

Quantitative Insights Into Microbial Ecology 2 (QIIME2) pipeline v.2021.11 [44] was applied to estimate bacterial and fungal populations. Firstly, adapter sequences were removed from raw reads using the cutadapt plugin [45]. Subsequently, using the DADA2 algorithm [46], PCR primers were removed from the reads and quality filtering was performed, followed by dereplication and denoising of the filtered reads. Chimeric sequences along with singletons were then removed from the data. Finally, paired sequences were merged, resulting in the final amplicon sequence variants (ASVs). For taxonomic classification, ASVs were aligned to SILVA 138 database [47] with 99% sequence similarity. At a final step, any archaeal, chloroplastic, mitochondrial, or unassigned sequences were removed from the bacterial ASV table. Respectively, any bacterial, unassigned, or eukaryotic, non-fungal sequences were removed from the fungal ASV table.

Visualization of ASV tables and .biom files was conducted in R version 4.2.1 [48]. ASV tables and .biom files were merged and imported into R environment using the phyloseq R package [49]. Alpha diversity indices (Shannon, Simpson, and inverse Simpson) were estimated for each sample using the ‘estimate_richness’ function of ampvis2 R package [50]. Total sum scaling method (TSS) was applied to 16S rRNA and 18S rRNA ASV tables; thus, bar plots were normalized to 100% as abundance estimations within each sample. Principal coordinates analysis (PCoA) was conducted using the ‘amp_ordinate’ command of ampvis2 package, by applying the Bray–Curtis distance method. Functions from ggplot2 R package [51] were combined with phyloseq and ampvis2 commands to generate bar plots and PCoA plots.

### 2.4. Biochemical Analysis

#### 2.4.1. Reagents and Chemicals

All reagents used were analytical-grade and HPLC-grade purity. Methanol, water, acetonitrile, and H_3_PO_4_ were obtained from Chem-Lab NV (Zeldegem, Belgium). Folin–Denis reagent, DPPH, and caffeic acid were purchased from Sigma-Aldrich (St. Louis, MO, USA). Oleuropein (CAS: 32619-42-4) and 3-hydroxytyrosol (CAS: 10597-60-1) standards were obtained from Tokyo Chemical Industry Co. (TCI, Tokyo, Japan). Oleocanthal (CAS: 289030-99-5) and oleacein (CAS: 149183-75-5) standards were purchased from Carbosynth (Compton, UK) and PhytoLab (Vestenbergsgreuth, Germany), respectively.

#### 2.4.2. Preparation of Samples and Extracts

Fifty (50) grams of each olive sample were placed in sterile disposable plastic containers in the presence of 5 mL of ddH_2_O and homogenized to a pulp using a polytron homogenizer (Polytron PT-MR 6100, Kinematica AG, Littau, Switzerland). The pulp was then frozen, lyophilized (LyoQuest, Telstar, Madrid, Spain), and pulverized.

Polyphenols were extracted from the pulverized sample using methanol, according to Boskou et al., 2006 [52]. Briefly, 0.5 g of dry sample was extracted five times with 5 mL methanol each time and the extracts were pooled and evaporated to dryness under nitrogen flow. The dry residue was reconstituted with 5 mL methanol and the methanolic extracts were used for the determination of total phenolics, antioxidant activity, oleuropein, oleocanthal, oleacein, and 3-hydroxytyrosol.

#### 2.4.3. pH Measurement

The measurement of each sample’s pH was made using the olive homogenate before lyophilization with a digital pH meter (C5010, Consort, Turnhout, Belgium).

#### 2.4.4. Total Polyphenol Content

Aliquots of the methanolic extracts were used for the determination of total phenolic content with Folin-Denis reagent according to the method described by Lanza et al., 2010 [53]. Total phenolics were expressed as mg of caffeic acid/100 g of dry olives. All the spectrophotometric data were acquired using a UV-2600 (Shimadzu, Kyoto, Japan) UV-Vis spectrophotometer.

#### 2.4.5. Antioxidant Activity

Brand-Williams method (DPPH‧ assay) [54] with some modifications was used to determine the antioxidant activity of the different extracts as described in Boskou et al., 2006. The results were expressed as EC50, which is the mg of polyphenols required to scavenge 50% of the initial concentration of the DPPH‧ reagent.

#### 2.4.6. Quantification of Oleuropein, Oleacein, Oleocanthal and 3-Hydroxytyrosol with HPLC

Oleuropein, oleacein, oleocanthal, and 3-hydroxytyrosol were quantified with a Dionex UltiMate 3000 HPLC-DAD apparatus (Thermo Fisher Scientific, Waltham, MA, USA), using a Hypersil GoldTM aQ C18 reverse-phase column (5 μm, 150 × 4.6 mm) (Thermo Fisher Scientific, Waltham, MA, USA). The identification of each compound was achieved by comparing its retention time with the retention time of a standard. The measurement of the compounds was performed according to the method of the International Olive Council (2017) with minor modifications. Briefly, the detection was performed at 280 nm, and the run was carried out at 26 °C. The solvents used as mobile phase were 0.2% H_3_PO_4_ in water as solvent A and 50% acetonitrile in MeOH as solvent B. The elution gradient was set up for solvent B as 4% to 50% in 40 min; 50% to 60% in 5 min; 60% to 100% in 15 min and hold for 10 min; and 100% to 4% in 2 min and hold for 10 min. The flow rate was 1 mL/min, and the run time was 82 min. The quantification of all compounds was determined by a calibration curve of each polyphenol standard ranging between 1 and 500 ppm, with a regression coefficient value (R) of 0.999.

#### 2.4.7. Statistical Analysis

For each biochemical analysis, triplicate measurements were conducted, and data were expressed as mean value ± standard error (*n* = 3). Statistical analysis was performed using unpaired *t*-test (GraphPad, San Diego, CA, USA), while *p*-value significance threshold was 0.05 (*p* ≤ 0.05).

## 3. Results

### 3.1. Amplicon Metabarcoding Analysis

#### 3.1.1. 16S rRNA Bacterial Assessment

For the characterization of the bacterial population of the samples of interest, the hypervariable regions V3–V4 of 16S rRNA gene were sequenced. In total, 897,320 raw reads were obtained, which were further reduced to 371,706 after quality- and taxonomy-based filtering (Table S1). On average, 20,650 quality-filtered reads per sample were obtained for the identification of bacterial populations. Microbial diversity was estimated using alpha diversity indices: number of observed ASVs, Shannon, Simpson, and inverse Simpson. Samples Kal_3 and Amf_2 had the highest number of observed ASVs (Table 2) as well as the highest Shannon index value, indicating high species richness. At the same time, Kal_1, Kal_2, and Halk_1 had the lowest number of observed ASVs, while Kal_1, Halk_2, Thas_1, and Kons_3 had the lowest Shannon index values, suggesting low diversity. Evaluation of the Simpson index, which gives more weight on species evenness within a sample, also revealed that the bacterial diversity was increased in the samples Amf_2, Kal_3, and Halk_6. In general, great variability regarding the detected species and their relative abundance was observed between samples of the same cultivar or treatment.

**Figure 1:**
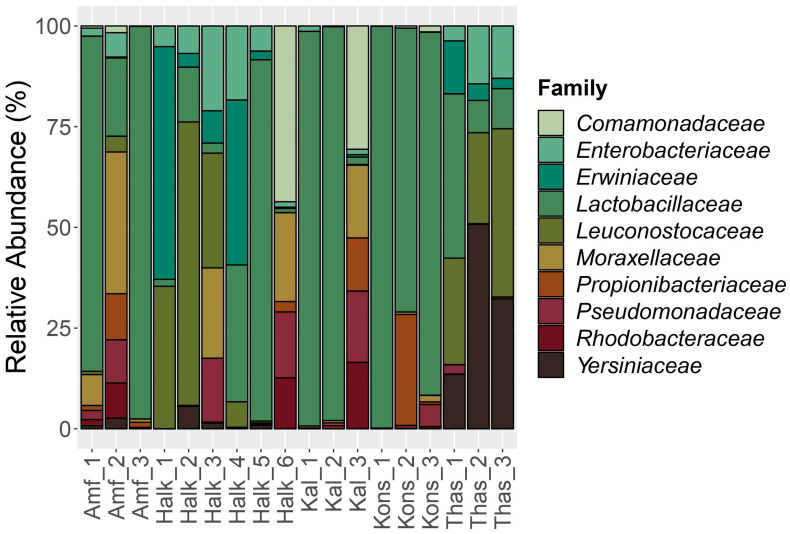
Distribution of the major bacterial families detected in the samples. The scale in the y-axis reflects the normalized relative abundance percentages (%).

In more details, Firmicutes were the dominant bacterial Phylum, ranging from 99.7% in relative abundance in Kons_1 sample to 16.7% in the sample Halk_6, followed by Proteobacteria, ranging from 71.0% (Halk_6) to 0.3% (Kons_1) (Table S2). Bacilli were the most abundant class, with a relative abundance of 61.0% on average, followed by Gammaproteobacteria (29.4%) and Alphaproteobacteria (3.5%). At the Order taxonomic level, the majority of the isolated bacteria belonged to Lactobacillales, 59.6% on average, followed by Enterobacterales (18.1%) and Pseudomonadales (6.2%). The prevailing bacterial taxa at the family level (Figure 1) were *Lactobacillaceae* (44.7% on average), followed by *Leuconostocaceae* (12.7% on average) and *Erwiniaceae* (7.2% on average). Samples Kons_1, Kal_1, Kal_3, and Amf_3 were the richest in *Lactobacillaceae*, with percentages above 96.0%, while all the other microbial populations in these samples were below 1.3%. Interestingly, Throuba Thassou and Throuba Halkidikis samples were the only ones not dominated by *Lactobacillaceae* family, on average, while *Leuconostocaceae* was the most abundant family in Throuba Halkidikis olives and the second most abundant in Throuba Thassou. Members of *Enterobacteriaceae*, *Yersiniaceae*, and *Erwiniaceae* were also in the most abundant families. Thas_1 sample had the highest percentage of LAB and the lowest of *Enterobacteriaceae*, while Thas_2 had the opposite trend.

Konservolia samples were on average the richest in *Lactobacillaceae* family bacteria. Bacteria of the *Comamonadaceae* family were also detected in samples Halk_6 and Kal_3. These two samples were the only samples stored in a jar in brine and shared common bacteria that were not detected in other samples. Apart from *Comamonadaceae* family, bacteria from the *Rhodobacteraceae* family were also in abundance in Halk_6 and Kal_3.

At the genus level, three genera dominated at similar percentages, *Lentilactobacillus*, *Leuconostoc*, and *Lactobacillus* (13.9%, 12.0%, and 11.6% on average, respectively), followed by *Pediococcus* and *Lactiplantibacillus. Lentilactobacillus* genus bacteria were absent from Throuba Thassou and Throuba Halkidikis. Other genera were also identified in our dataset but in much lower relative abundances; on average, *Erwinia* (4.6%), *Serratia* (5.6%), *Acinetobacter* (2.9%), *Pseudomonas* (2.7%), and *Alicycliphilus* (2.3%) completed the 10 most abundant bacterial genera. At the species level, several members of the *Lactobacillaceae* family were identified, such as *Lactiplantibacillus pentosus*, *Lentilactobacillus buchneri*, *Leuconostoc pseudomesenteroides*, *Pediococcus ethanolidurans*, *Leuconostoc mesenteroides*, and *Lentilactobacillus parafarraginis*. The 10 most abundant species also included *Rahnella* sp. The bacterium *Candidatus Erwinia dacicola*, an olive fly endosymbiont [55], was included in the 15 most abundant species as it was found in high abundance (40.0%) in the sample Halk_4, together with *Lentilactobacillus parafarraginis*, *Pediococcus parvulus*, *Enterobacteriaceae* bacterium, and *Hafnia* sp.

Spoilage organisms or potential pathogens, such as *Enterobacteriaceae*, *Pseudomonadaceae*, *Listeriaceae*, *Clostridium*, and *Vibrionaceae*, were detected in very low percentages in all samples. In more details, bacteria of the family *Enterobacteriaceae* were detected in trace amounts in all the samples except for Konservolia cultivar, and in high relative abundance in Halkidikis and Thassou samples (11.2–18.0%), represented by the species *Enterobacter cloacae*, *Enterobacter ludwigii*, *Kluyvera cryocrescens*, *Klebsiella aerogenes*, *Lelliottia* sp., and *Kosakonia oryzae*. *Brochothrix thermosphacta* was the only member of *Listeriaceae* family found in Amf_1, Amf_2, Halk_4, and Kal_2 samples, whereas *Pseudomonas stutzeri* was identified in very low abundance in samples from all the cultivars, except for Throuba Thassou. The Amfissis samples had traces of bacteria (0.3–1.5%) belonging to *Vibrionaceae* family, Amf_2 in higher percentage. *Clostridiaceae* family was represented by the species *Clostridium beijerinckii*, which was found in samples Amf_1, Amf_2, Halk_5, Halk_6, and Kon_3, whereas the well-characterized foodborne pathogens *Clostridium botulinum* and *Clostridium perfringens* [56] were not found in any of the samples.

**Figure 2:**
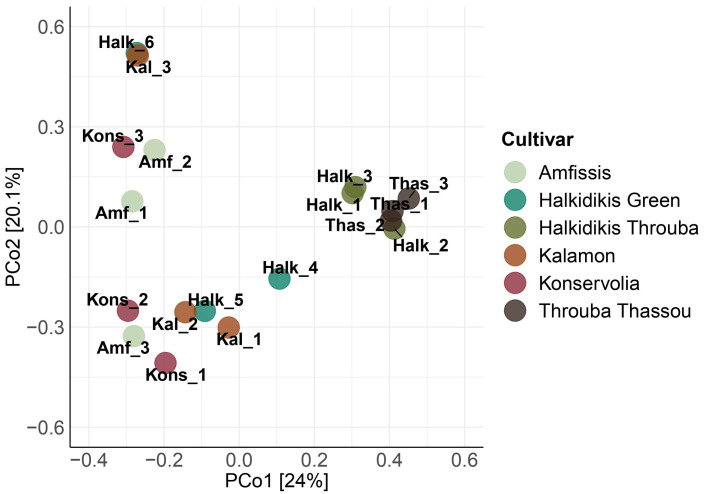
Principal coordinates analysis (PCοA) for the 16S rRNA amplicon metabarcoding data for all samples.

Principal coordinates analysis (PCoA) of the 16S rRNA data (Figure 2) revealed the formation of some clusters. All Throuba Thassou samples clustered together along with Halk_2, while in close proximity, Halk_1 and Halk_3 formed another cluster. Interestingly, samples Halk_6 and Kal_3 formed a tight cluster in distance from all the other samples. The rest of the table olives did not show any clear grouping, although Halk_5 and Kal_2 were very close to each other but also in close proximity to the rest of the samples.

#### 3.1.2. 18S rRNA Eukaryotic Assessment

To assess the eukaryotic community of the different samples, the V7–V8 hypervariable regions of the 18S rRNA gene were analyzed, as taxonomic identification markers. In total, 1,221,185 raw reads were obtained, corresponding on average to 67,843 raw reads per sample (Table S1). After quality and taxonomic filtering, these reads were reduced to 524,901, with an average of 29,161 reads per sample.

The sample Kal_3 had the highest number of observed ASVs, followed by Halk_6, while Kons_1 had the lowest (Table 3). Sample Kal_3 and three samples from Halkidikis cultivar, 3, 5, and 6, had the highest Shannon and Simpson indices, indicating high species richness and evenness.

The eukaryotic community was dominated by species belonging to the Fungi kingdom. Diversity between the samples appears at the family level, where 11 families occupied the various samples (Table S3**)**. In more details, *Pichiaceae* was the prevailing family with an average abundance of 23.8%, dominating 5 out of 18 samples (Amf_3, Halk_5, Kal_2, Kons_1, and Kons_3), while *Phaffomycetaceae* was the second most abundant (14.9%), present in high amounts in samples Halk_2, Kons_2, and Thas_2 (90.8%, 72.1%, and 63.5%, respectively). *Saccharomycetaceae* was the third most abundant family (12.4% on average), while *Aspergillaceae*, containing mold species, was fourth in relative abundance (10.3%), dominant in Amf_1 (98.7%) and Thas_1 (42.8%). The top 10 of the most abundant families are completed with *Debaryomycetaceae*, *Pleosporaceae*, *Aureobasidiaceae*, *Malasseziaceae*, *Cladosporiaceae*, and *Dipodascaceae*. The taxon that was classified as *incertae sedis* within fungi included members of the Starmerella-Candida clade.

**Figure 3:**
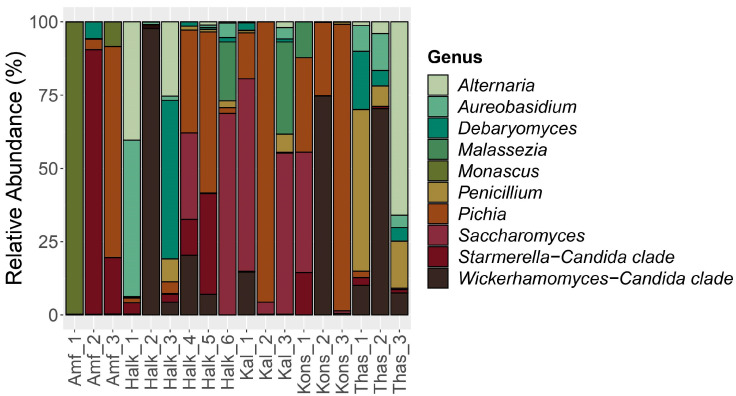
Distribution of the major fungal genera detected in the samples. The scale in the y-axis reflects the normalized relative abundance percentages (%).

Great biodiversity between samples of the same cultivar was observed at the genus level (Figure 3). For instance, *Monascus,* a mold, was the most abundant genus in Amf_1 (98.7%), while species belonging to Starmerella-Candida clade were abundant in Amf_2 (83.1%) and yeasts of the genus *Pichia* in Amf_3 (65.3%). Interestingly, the six samples from Halkidiki demonstrated different eukaryotic communities prevailing in their load. In Kal_1 and Kal_3 samples, the dominant genus was *Saccharomyces*, while in Kal_2 it was *Pichia*. In Konservolia samples, the genera *Brettanomyces*, Wickerhamomyces-Candida clade, and *Pichia* prevailed. In Throuba Thassou, the prevailing fungi in each sample belonged to the genera *Penicillium*, Wickerhamomyces-Candida clade, and *Alternaria*, respectively. On average, *Pichia* was the most abundant genus (19.4%), followed by Wickerhamomyces-Candida clade (14.9%), *Saccharomyces*, and Starmerella-Candida clade, both at 8.1%. Samples Halk_6 and Kal_3 were dominated by *Saccharomyces*, while the second most popular genus was *Malassezia* at 12.1% and 17.3% abundance, respectively, that was not detected in any other sample except from Kons_1 at 2.6%.

At the species level, great variability was observed between the samples, as a consequence of the diversity at the genus level. In sample Amf_1, the genus *Monascus* was represented by *Monascus ruber* (98.7%), which was found in very low abundance in the other two Amfissis samples (0.1% and 7.6%, respectively). In Amf_2, the main species was *Starmerella etchellsii*, while in Amf_3 it was *[Candida] ethanolica*, which was only in 0.0–0.2% abundance in the other two Amfissis samples. The samples from Halkidikis cultivar also had distinct prevailing species. For instance, the dominant species in Halk_1 was *Aureobasidium pullulans* (51.1%), which was found at below 3.0% in the rest of the samples. Halk_2 was dominated by *Wickerhamomyces anomalus* (90.8%), Halk_3 by *Debaryomyces hansenii* (36.4%), and Halk_4 by *Zygotorulaspora mrakii* (53.0%). In the last two samples, Halk_5 and Halk_6, the prevailing species were *Pichia membranifaciens* (31.3%) and *Saccharomyces cerevisiae* (41.4%), respectively. In Kal_1 and Kal_3 samples, the dominant species was also *Saccharomyces cerevisiae*, while in Kal_2 it was *[Candida] ethanolica*. *Alternaria* species were found in abundance in samples Halk_1 (38.6%), Halk_3 (16.8%), and Thas_3 (56.2%). The three Konservolia samples were populated mainly by *Brettanomyces custersianus*, *Wickerhamomyces anomalus*, and *Pichia membranifaciens*, respectively. In Thas_1, the highest percentage was of the species *Penicillium chrysogenum* (42.8%), in Thas_2 it was *Wickerhamomyces anomalus* (63.5%), while in Thas_3 it was *Clathrospora diplospora*. The fungus *Phoma* sp. belonging to *Didymellaceae* family, that is related with debittering of the Throuba Thassou olives on the tree but is also classified as phytopathogenic [57], was detected in trace amounts only in the sample Halk_6.

**Figure 4:**
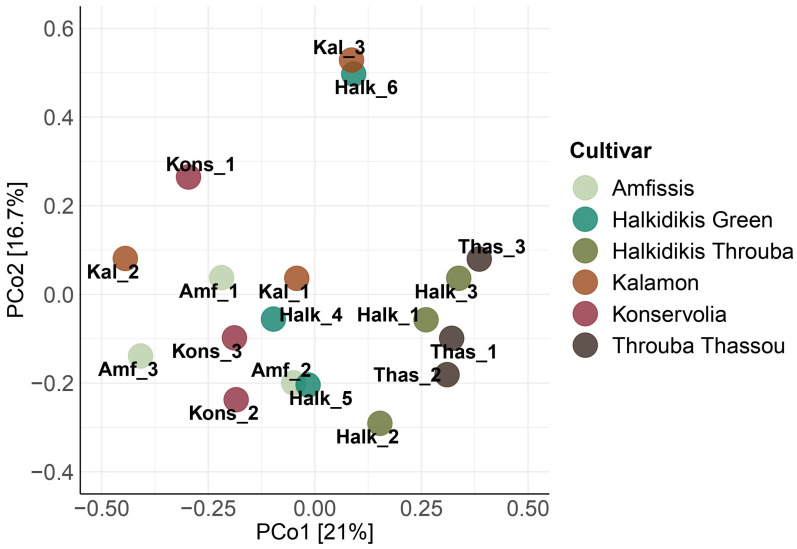
Principal coordinates analysis (PCoA) for the 18S rRNA amplicon metabarcoding data for all samples.

Principal coordinates analysis (PCoA) of the 18S rRNA data (Figure 4) revealed a grouping of Kal_3 with Halk_6 in distance from the rest of the samples, as it was observed in 16S rRNA PCoA analysis before. All Throuba Thassou samples along with Halk_1 and Halk_3 samples were closer to each other than to the rest of the samples, forming a loose cluster, with Halk_2 in close proximity. The rest of the samples did not show a clear pattern of clustering, with the exception of a notable closeness of Amf_2 with Halk_5.

### 3.2. Biochemical Analysis

**Figure 5:**
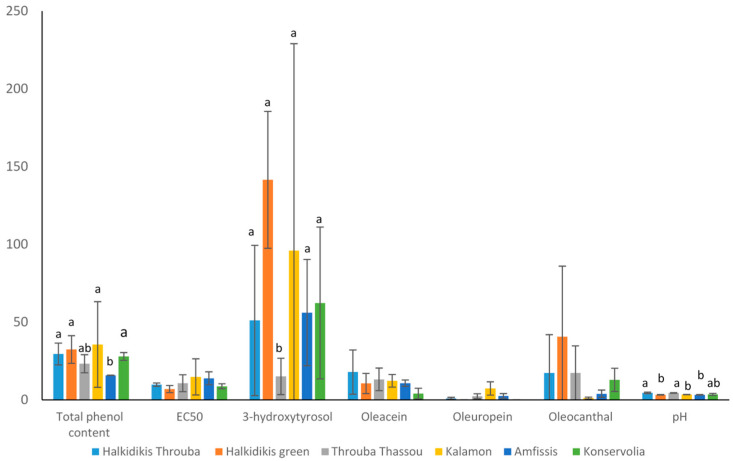
Biochemical values of the samples, expressed as average for each cultivar. Units of measurement are total phenol content (mg of caffeic acid/10 g of dry olives), 3-hydroxytyrosol, oleacein, oleuropein, oleocanthal (mg/100 g of dry olives). A statistically important difference (*p* ≤ 0.05) is marked with a different letter.

Biochemical analysis using various methodologies highlighted the nutritional value of each sample (Table 4). The pH value of each sample was also assessed (Table S4) revealing that the average value of Throuba Thassou (4.5) and Throuba Halkidikis (4.5) was significantly higher than the average value of Halkidikis green (3.3), Kalamon (3.4), and Amfissis olives (3.3). Konservolia samples had an intermediate pH value of 3.6 (Figure 5). Regarding the phenol content, the table olives Halk_6 and Kal_3 had the highest, while Kal_1 and Thas_2 had the lowest. Consequently, antioxidant activity exhibited a reversed pattern where Kal_1 had the highest while Kal_3 had the lowest EC50 value.

**Figure 6:**
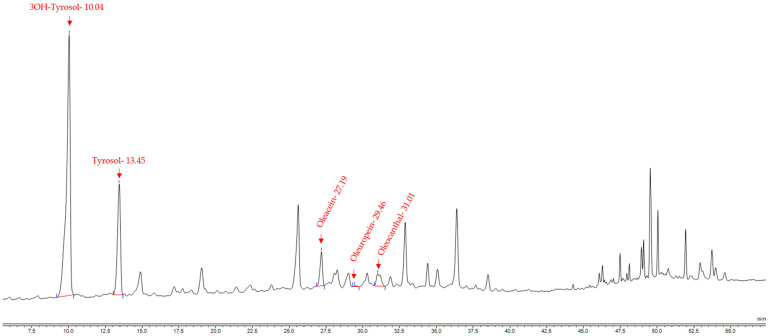
HPLC chromatogram of sample Kal_3, indicating with red letters all the different compounds identified. Hydroxytyrosol (3OH-Tyrosol), retention time 10.04 min; tyrosol, retention time 13.45 min; oleacein, retention time 27.19 min; oleuropein, retention time 29.46 min; oleocanthal, retention time 31.01 min.

Hydroxytyrosol, oleuropein, oleocanthal, and oleacein values were interpreted from the HPLC chromatogram of each sample, as seen in Figure 6 for sample Kal_3. Hydroxytyrosol levels ranged from 6.3 to 249.3 mg/100 g of olives (or 63–2493 ug/g), observing the highest values in Kal_3, Halk_6, and Halk_4, while the lowest were observed in Thas_1 and Thas_2 samples.

Oleacein reached its maximum levels in samples Halk_2 and Thas_2, both samples treated by salt-drying. Oleuropein levels were the lowest compared to other metabolites, ranging from undetectable to 7.4 mg/100 g (or 74 ug/g). In the majority of the samples, 12 out of 18, oleuropein could not be detected, regardless of the debittering process. In the rest of the samples, the highest amount was observed in Kal_2 and Thas_3. The highest oleocanthal levels were reported in two samples of Halkidikis, one green and one black, as well as in one Thassou sample. Figure 5 illustrates the results as calculated per cultivar. The samples of Halkidikis cultivar were divided into two groups, based on the color of the fruit and the treatment. When the biochemical results were interpreted as average per cultivar, it was observed that Spanish-style green olives had the highest levels of phenol content, followed by salt-dried olives; however, their difference was not statistically significant. The phenol content was significantly lower in Amfissis cultivar compared to Kalamon olives, which were both Greek-style black olives.

Regarding 3-hydroxytyrosol, Throuba Thassou had significantly lower levels compared with Halkidikis green olives. The rest of the groups did not differ significantly; however, great variation within the same cultivar was observed, especially in Kalamon olives where the last sample had 10- and 20-fold higher levels than the others. Regarding the rest of the values measured, no statistically significant difference was observed between the cultivars, as great difference was observed within each cultivar. For instance, when measuring oleocanthal, responsible for olives’ pungent taste, sample Halk_4 had the highest value, while in Halk_5 it was 40-fold lower and in Halk_6 it was not even detected (Table 4).

## 4. Discussion

Table olives are among the most popular fermented foods worldwide. They have a unique texture and complexity of flavor, while their consumption offers great health benefits. Due to the natural bitterness of the olive fruit, table olives undergo several processes to become edible. These treatments affect the microbiome of the final product, which greatly contributes to the taste and organoleptic properties of the table olives. Thus, the determination of the microbial communities present on table olives is of great importance for the producers. Several studies have elucidated table olives microbiome but in regard to their packaging and storage conditions [32,58,59]. Our work focuses on describing and comparing microbial communities based on relative abundance of taxa, while further statistical analysis could reinforce our observations.

In green Spanish-style Halkidikis olives, studies have shown the prevalence of the bacteria *Lactiplantibacillus pentosus* and *Pediococcus ethanolidurans* and the yeast *Pichia membranifaciens* on the fruit and in the brine [9,10,60]. Our green Spanish-style Halkidikis samples, Halk_4 and Halk_5, followed the same pattern, as they were dominated by *Saccharomycetaceae* and *Pichiaceae* families, and the above-mentioned species were detected. Sample Halk_6 did not have the bacterial community described above as it was dominated by bacteria of *Rhodobacteraceae* and *Comamonadaceae* families. Bacteria of *Comamonadaceae* family are typically discovered in olive mill wastewaters [61] and are considered symbionts of olive fruit flies [62], while bacteria from the genus *Paracoccus*, the identified genus of *Rhodobacteraceae* in Halk_6, have been isolated from green Spanish-style Manzanilla olives brine [60]. It is interesting to mention that Halk_6 was the only sample from Halkidikis cultivar where the olives were packed in brine in a jar, same as sample Kal_3 that was the only Kalamon sample in brine in a jar. Kal_3 sample shared the same bacterial community profile as Halk_6; as a result, these samples were in very close proximity in PCoA of 16S data (Figure 2), while Kal_1 and Kal_2 were closer together, same as Halk_4 with Halk_5. Kons_3 olives, that were also stored in brine but in a plastic container under MA, exhibited a microbial community composition more similar to samples packed in MA. This suggests that the application of modified atmosphere plays a crucial role in shaping the microbial community of the final product. The storage in brine in a jar seems to have an additional effect on the fungal community of Halk_6 and Kal_3, since it was observed that they shared a unique species belonging to *Malassezia* genus. *Malassezia* genus comprises of lipophilic species that are part of the normal human skin [63], implying that there could have been a contamination during olive processing. There is one study where yeast belonging to Malasseziales order was found on olive tree twigs of Madural cultivar [64], implying that the species found in our samples originated from the olive fruit itself, while another study has reported its presence in green and black natural table olives of various cultivars [65].

Regarding Kalamon cultivar, studies have shown that black Kalamon olives fermented in brine were dominated by *Lactiplantibacillus*, *Celerinatantimonas*, *Propionibacterium*, and *Pseudomonas* species [30,66]. Other studies detected no LAB or *Enterobacteriaceae* in the final product, whereas the yeasts *Candida boidinii*, *C. molendinolei, Pichia manshurica*, and *S. cerevisiae* dominated the table olives at the end of fermentation [29]. Other researchers found that the dominant yeast species in black Kalamon table olives were *Saccharomyces cerevisiae*, *Pichia anomala*, *Debaryomyces hansenii*, *P. membranifaciens*, and *Guehomyces pullulans* [13], while others reported *Pichia*, *Candida*, *Wickerhamomyces*, and *Citeromyces* species [65]. The yeasts found in Kalamon samples of this study, *Saccharomyces cerevisiae*, *[Candida] ethanolica*, and *Wickerhamomyces anomalus*, synonymous for *Pichia anomala*, are in accordance with the aforementioned studies. Although Bonatsou et al., 2018 detected no LAB in the final product of Kalamon olives, numerous other studies reported their dominance [13,30,32,58,66] as it was observed in our samples. *Enterobacteriaceae* were detected in amounts below 1.3% and could potentially affect the sensorial quality of the product since they are implicated in the formation of gas pockets [12,67].

As previously mentioned, in the literature, Konservolia is most of the times identical to Amfissis cultivar; however, cultivars from other areas of Greece may be referred to as Konservolia. We chose to separate the Amfissis from Konservolia samples, since for the latter the cultivation area was not mentioned on the package. In the literature, *Phaffomycetaceae*, and more specifically *Wickerhamomyces anomalus*, as well as *Pichiaceae* with *Pichia membranifaciens*, were the dominant yeasts identified in Konservolia samples [9,65]. In another study, using a culture-dependent method, Bleve et al., 2015 identified the yeast species *Debaryomyces hansenii* and *Pichia anomala* in black Konservolia table olives fermented by Greek-style [13]. Samples Kons_2 and Kons_3 were indeed dominated by *Wickerhamomyces anomalus* and *Pichia membranifaciens*, respectively, while the most abundant species in Kons_1 was *Brettanomyces custersianus*, which has been detected in Halkidikis green olives and in Kalamon black olives [9,32]. In our study, this species was identified only in Konservolia samples. *Debaryomyces hansenii* was the most abundant yeast in Halk_3 and it was detected in lower amounts in all Throuba Thassou samples, meaning that in our study it was detected only in salt-dried olives.

*Monascus ruber*, that dominated Amf_1, is a mold producing red pigments, used traditionally for coloring of food and wine [68]. It has been isolated from Konservolia olives in other studies [69,70] and is classified as a spoilage microorganism. Tzamourani et al., 2021 reported the lack of *Enterobacteriaceae* and the prevalence of *Pediococcus ethanolidurans* and *Pichia membranifaciens* in the final product of green Spanish-style Konservolia table olives [10]. Additionally, in our study, no *Enterobacteriaceae* were detected neither in Konservolia nor in Amfissis samples, while *Pediococcus ethanolidurans* was the dominant bacterium in Konservolia samples and the third most abundant in Amfissis samples.

According to studies, dry-salted table olives from Throuba Thassou cultivar were dominated by yeasts, while bacterial populations were absent [24]. In black dry-salted olives of Manzanilla cultivar, LAB were not found in significant amounts, while *Enterobacteriaceae*, yeasts, and mold were the dominant microbes [71]. The same populations, *Enterobacteriaceae*, other mesophilic aerobic bacteria, yeasts, and absence of lactobacilli were also reported in the fresh fruits of the Turkish cultivar Hurma, that is left to debitter naturally on the tree, such as Throuba olives [72]. The absence of LAB in Throuba Thassou was again reported by Panagou et al., 2002, where the microbial flora was composed of yeasts [73]. In our Throuba Halkidikis and Throuba Thassou samples, the detected percentage of *Lactobacillaceae* family was low; however, *Leuconostocaceae* was the most abundant family in Throuba Halkidikis olives and the second most abundant in Throuba Thassou, as presented in Figure 1. Only in sample Thas_2, lactobacilli were not the most abundant bacterial population, and it is worth mentioning that it was the only sample packed under MA.

*Alternaria* species, which were found in abundance in samples Halk_1, Halk_3, and Thas_3, are major plant pathogens isolated from a wide range of food products, such as olives as well as their derived products [65,74]. Notably, the three samples where *Alternaria* was found in abundance were black olives left on the tree until full maturation, and therefore were exposed to pathogens for a greater period of time. The presence of this fungus on the olive does not necessarily mean that the mycotoxins it produces will be found in the final product [75]; however, biochemical analysis may be valuable.

It is worth mentioning that none of the well-characterized foodborne pathogen bacteria (*Bacillus cereus*, *Campylobacter jejuni*, *Clostridium botulinum*, *Clostridium perfringens*, *Cronobacter sakazakii*, *Esherichia coli*, *Listeria monocytogenes*, *Salmonella* spp., *Shigella* spp., *Staphylococcus aureus*, and *Yersinia enterocolitica*) [56] were detected in the analyzed samples, with the exception of an uncultured bacterium of *Vibrio* genus found in one of the Amfissis samples at 1.5% abundance. *Vibrio* species have been found in low percentages in various types of table olives in the market [66]. The species found in our sample has a 100% match of sequence with *Vibrio hibernica* and *Vibrio rumoiensis*. These two species have been reported to have very high genome similarity [76]. *Vibrio hibernica* has been found in fermented foods, not causing spoilage but actually having industrial applications in food processing [76], while the source of it may be the salt used for the brine [77]. *Vibrio rumoiensis* has been isolated from packed ham [78] and salted fish [79].

*Brochothrix thermosphacta*, that was in the top 20 bacteria found due to its presence in the sample Amf_2, in which it was the dominant bacterium, and in Amf_1 in lower percentage, is characterized as a food spoilage organism mainly found in meat products, causing off-odors [80], and it has been found on Spanish-style, green and black natural table olives in trace amounts [66]. In these two samples, Amf_1 and Amf_2, various potential spoilage or even pathogenic microbes were discovered (*Vibrio* sp., *Clostridium* sp., *Brochothrix thermosphacta*), and as a result, they were in close proximity in 16S rRNA PCoA analysis (Figure 2). It is interesting that the samples Amf_1, Amf_2, and Amf_3 were processed by the same producer, but Amf_2 table olives were baked in the oven after fermentation, while Amf_1 and Amf_3 only differed in the fruit size and were packed right after debittering. The sample Amf_2 had the highest relative abundance of potential spoilage or pathogenic bacteria, which could mean that the extra step of baking introduced undesirable microorganisms. This could be attributed to the fact that the microbiome of a food may become affected by the processing environment, from the microbes present in the production line, the equipment, and the air [81].

The clustering of all the salt-dried olives on the PCoA analysis of the 16S rRNA (Figure S1) data implies that the salt-drying method offered a unique bacterial profile to the final product. The same cannot be concluded for the Spanish-style or Greek-style methods, as samples from both treatments were mixed in the various groups formed. This is related to the variability of species and abundances observed in samples of the same cultivar. Benitez-Cabello et al., 2020, also reported the absence of a distinct clustering between samples of Spanish-style, green natural and black natural table olives [66]. The same observation can be made for the PCoA analysis of the 18S rRNA data (Figure S2), concluding that the salt-drying method seems to additionally offer a unique fungal profile.

There are numerous factors that affect the phenolic content of table olives including the cultivar, degree of ripening, and, most importantly, the method used for fermentation and processing. Regarding the total phenol content, the observation of the highest (Kalamon) and lowest (Amfissis) levels within the same fermentation style, the Greek-style, suggests that in the specific samples, the phenolic content was more affected by the cultivar than the treatment. Kalamon olives have been reported in the past to have higher total phenols than Konservolia, Throuba Thassou, and Halkidikis green olives [82]. In other studies, where olive fruits from one cultivar were treated either by Spanish or Greek style, it was found that the phenolic content was higher in Greek-style olives [83]. The antioxidant capacity did not vary significantly between the samples analyzed in this study; thus, it was not clarified if it depends on cultivar or treatment style.

More than one study has reported that Spanish-style green olives and Greek-style black olives have similar levels of hydroxytyrosol, which was the predominant compound of all the phenolics [82,84]. Our samples were in accordance with this observation, since the only statistically important difference was observed between Throuba Thassou and Halkidikis green olives. This may be related to the fact that they show the opposite trend in oleuropein levels and it is known that oleuropein degradation results in the simpler molecule of hydroxytyrosol [85]. Low levels of hydroxytyrosol in Throuba Thassou olive variety compared to the cultivars included in our study were also shown by Blekas et al., 2002.

Another study of Greek table olive cultivars reported oleuropein content ranging from undetectable amounts up to 550 ug/g of olive flesh for Throuba Thassou and hydroxytyrosol content at 13.5–555.0 ug/g of olive flesh for Kalamon olives [21]. In our study, the oleuropein levels were lower, ranging from undetectable to 74 ug/g of olive flesh for Kalamon cultivar, while hydroxytyrosol levels were higher, ranging from 63 to 2493 ug/g of olive flesh, with the maximum levels observed in Kalamon black olives, in agreement with other studies [82].

Oleocanthal levels are considered an indicator of the bitter taste of table olives [86]; therefore, the results suggest that Halkidikis green olives had the most bitter taste, while Kalamon had the mildest. This is in accordance with observations in Manzanillo olives treated in Spanish style compared to Kalamon olives processed in Greek style [84]. Since oleocanthal levels were at their lowest values in Kalamon and Amfissis olives, it is implied that the Greek-style treatment resulted in a final product with less bitter taste than the rest of the varieties. Oleacein, a derivative of oleuropein degradation, contributes to the bitter taste of table olives and its levels are found to be very low, if not undetectable, in the final product, regardless of the fermentation style [84]. Our findings are in accordance with this observation.

Regarding the pH values measured, salt-dried olive units were in accordance with some studies [87] and a bit lower than others [24], while the lower pH values of the other treatments were expected according to [4,30,60,66].

It has been stated that table olives with high concentrations of oleuropein and related phenolics will have lower levels of LAB in fermentation brines, and yeast growth will dominate [86]. This could be observed in the samples Kal_3 and Halk_6 that had the highest values of total phenol content and 3-hydroxytyrosol and their bacterial community composition was significantly different from the other two samples of their group, showing a decrease in LAB (Figure 1). Moreover, LAB produce lactic acid, causing a drop in the pH of the final product [88], as it was observed in our study, since the salt-dried olives had a lower percentage of LAB and higher units of pH.

## 5. Conclusions

This study presents the analysis of the microbiome of six different Greek table olive varieties, along with the biochemical properties of the final product. A variety of bacteria and fungi were identified, indicating that the salt-drying fermentation style offered a unique microbial profile that distinguished Throuba Thassou and Throuba Halkidikis olives from Greek-style black olives and Spanish-style green olives. These last two types of olives could not be differentiated based solely on their bacterial and fungal profiles. The biochemical values showed great fluctuation in samples within the same cultivar/treatment, suggesting that each production line and packaging type greatly affected the final product. To the best of our knowledge, this is the first time that microbiome sequencing coupled with biochemical analysis has been performed in six different table olive varieties, providing useful information for producers and consumers.

## Figures and Tables

**Table 1:** Description of the samples studied. MA; modified atmosphere.

Sample Name	Cultivar	Region	Olive Color	Fermentation Type
Halk_1	Halkidikis (unpacked)	Halkidiki	Black	Salt-dried
Halk_2	Halkidikis (unpacked)	Halkidiki	Black	Salt-dried
Halk_3	Halkidikis (unpacked)	Halkidiki	Black	Salt-dried
Halk_4	Halkidikis (unpacked)	Halkidiki	Green	Spanish-style
Halk_5	Halkidikis (unpacked)	Halkidiki	Green	Spanish-style
Halk_6	Halkidikis (packed in brine, jar)	Halkidiki	Green	Spanish-style
Thas_1	Throuba Thassou (unpacked)	Thassos	Black	Salt-dried
Thas_2	Throuba Thassou (packed in MA, pouch)	Thassos	Black	Salt-dried
Thas_3	Throuba Thassou (unpacked)	Thassos	Black	Salt-dried
Kal_1	Kalamon (packed in MA, pouch)	Messinia	Black	Greek-style
Kal_2	Kalamon (packed in MA, pouch)	Messinia	Black	Greek-style
Kal_3	Kalamon (packed in brine, jar)	Messinia	Black	Greek-style
Amf_1	Amfissis (packed in MA, pouch)	Central Greece	Black	Greek-style
Amf_2	Amfissis (packed in MA, pouch)	Central Greece	Black	Greek-style
Amf_3	Amfissis (packed in MA, pouch)	Central Greece	Black	Greek-style
Kons_1	Konservolia (packed in MA, pouch)	Central Greece	Green	Spanish-style
Kons_2	Konservolia (packed in MA, pouch)	Central Greece	Green	Spanish-style
Kons_3	Konservolia (packed in brine under MA, plastic container)	Central Greece	Green	Spanish-style

**Table 2:** Number of ASVs (amplicon sequence variants) and alpha diversity indices for each sample based on 16S rRNA amplicon sequencing.

Sample Name	Observed ASVs	Shannon	Simpson	Inverse Simpson
Halk_1	34	2.32	0.84	6.11
Halk_2	44	1.80	0.62	2.61
Halk_3	95	3.53	0.94	17.27
Halk_4	49	2.57	0.82	5.47
Halk_5	104	3.39	0.94	16.01
Halk_6	361	4.01	0.95	21.67
Thas_1	57	2.17	0.78	4.50
Thas_2	44	2.45	0.85	6.78
Thas_3	39	2.48	0.84	6.44
Kal_1	26	1.67	0.68	3.10
Kal_2	36	2.28	0.83	5.77
Kal_3	469	4.48	0.97	34.50
Amf_1	261	3.56	0.88	8.53
Amf_2	456	5.10	0.98	56.15
Amf_3	82	3.20	0.93	15.11
Kons_1	47	2.27	0.78	4.51
Kons_2	78	2.95	0.91	10.58
Kons_3	131	2.18	0.72	3.52

**Table 3:** Number of ASVs (amplicon sequence variants) and alpha diversity indices for each sample based on 18S rRNA amplicon sequencing.

Sample Name	Observed ASVs	Shannon	Simpson	Inverse Simpson
Halk_1	29	1.22	0.59	2.44
Halk_2	24	0.45	0.17	1.21
Halk_3	52	2.38	0.82	5.69
Halk_4	20	1.63	0.68	3.17
Halk_5	29	2.15	0.83	5.98
Halk_6	54	2.58	0.82	5.41
Thas_1	35	2.10	0.77	4.44
Thas_2	22	1.42	0.58	2.37
Thas_3	41	1.73	0.66	2.92
Kal_1	25	1.54	0.67	3.05
Kal_2	16	0.92	0.38	1.62
Kal_3	99	2.86	0.88	8.08
Amf_1	12	0.10	0.03	1.03
Amf_2	26	1.08	0.43	1.77
Amf_3	20	1.35	0.57	2.35
Kons_1	10	1.48	0.68	3.08
Kons_2	27	0.95	0.46	1.84
Kons_3	22	0.34	0.11	1.12

**Table 4:** Biochemical values of the 18 samples analyzed. The results are expressed as mean ± standard deviation (*n* = 3). Units of measurement for total phenol content is mg of caffeic acid/100 g of dry olives, while for 3-hydroxytyrosol, oleacein, oleuropein, and oleocanthal it is mg/100 g of dry olives. N.D., not detected.

Sample Name	Total Phenol Content	EC50	3-Hydroxytyrosol	Oleacein	Oleuropein	Oleocanthal
Halk_1	256.57 ± 18.21	9.92 ± 1.48	106.66 ± 0.85	11.63 ± 0.03	N.D.	1.66 ± 0.14
Halk_2	375.53 ± 7.73	8.74 ± 0.72	27.12 ± 0.66	34.19 ± 0.69	1.15 ± 0.04	45.74 ± 0.64
Halk_3	253.42 ± 10.13	10.83 ± 1.27	19.47 ± 0.43	7.86 ± 0.56	0.99 ± 0.13	4.71 ± 0.23
Halk_4	297.60 ± 0.94	5.07 ± 0.40	143.68 ± 3.75	6.94 ± 0.09	N.D.	79.43 ± 1.12
Halk_5	250.75 ± 4.88	9.50 ± 0.31	96.46 ± 1.38	6.75 ± 0.17	N.D.	1.90 ± 0.14
Halk_6	422.79 ± 6.33	6.39 ± 0.16	184.31 ± 6.09	18.05 ± 0.93	N.D.	N.D.
Thas_1	278.43 ± 13.84	8.69 ± 1.10	10.72 ± 0.06	8.70 ± 0.23	N.D.	3.41 ± 0.53
Thas_2	166.99 ± 0.27	6.63 ± 0.45	6.31 ± 0.72	21.53 ± 0.04	1.59 ± 0.16	11.50 ± 0.35
Thas_3	250.27 ± 0.96	16.84 ± 0.58	28.32 ± 1.38	9.37 ± 0.94	3.20 ± 0.38	36.93 ± 0.30
Kal_1	125.79 ± 0.04	26.46 ± 0.41	11.45 ± 0.18	9.65 ± 0.72	N.D.	1.14 ± 0.05
Kal_2	282.09 ± 8.14	14.68 ± 0.89	27.27 ± 0.80	10.31 ± 0.57	7.36 ± 0.23	N.D.
Kal_3	660.61 ± 1.51	3.29 ± 0.47	249.33 ± 20.58	16.97 ± 1.59	N.D.	N.D.
Amf_1	167.47 ± 0.28	9.10 ± 0.37	91.93 ± 1.90	8.81 ± 0.73	N.D.	3.98 ± 0.17
Amf_2	149.42 ± 5.46	16.90 ± 0.33	52.38 ± 2.56	10.69 ± 0.35	2.59 ± 0.43	N.D.
Amf_3	157.03 ± 3.34	15.68 ± 1.11	24.04 ± 0.15	12.88 ± 0.82	N.D.	N.D.
Kons_1	267.26 ± 7.88	10.48 ± 0.33	14.17 ± 0.14	6.49 ± 0.15	N.D.	12.86 ± 0.33
Kons_2	261.25 ± 2.09	8.38 ± 0.44	111.70 ± 0.38	1.65 ± 0.04	N.D.	N.D.
Kons_3	308.19 ± 8.11	7.48 ± 0.15	60.95 ± 3.63	N.D.	N.D.	N.D.

## Data Availability

The data are available from the corresponding author.

## Supplementary Materials

The following supporting information can be downloaded at: https://www.mdpi.com/article/10.3390/foods12071527/s1, Figure S1: Principal coordinates analysis (PCοA) for the 16S rRNA amplicon metabarcoding data for all samples according to their packaging; Figure S2: Principal coordinates analysis (PCοA) for the 18S rRNA amplicon metabarcoding data for all samples according to their packaging; Table S1: Number of raw and retained reads per sample after filtering for 16S and 18S rRNA sequencing results; Table S2: Table of abundances of amplicon sequence variants (ASVs) for 16S rRNA sequencing data; Table S3: Table of abundances of amplicon sequence variants (ASVs) for 18S rRNA sequencing data; Table S4: pH value of all olive samples; measurements were made in triplicates. SD, standard deviation.

## References

[1] Loumou A., Giourga C. (2003). Olive Groves: “The Life and Identity of the Mediterranean”. Agric. Hum. Values.

[2] International Olive Council Table Olives Consumption. https://www.internationaloliveoil.org/wp-content/uploads/2020/12/OT-W901-23-11-2020-C.pdf.

[3] Tassou C.C., Panagou E.Z., Katsaboxakis K.Z. (2002). Microbiological and Physicochemical Changes of Naturally Black Olives Fermented at Different Temperatures and NaCl Levels in the Brines. Food Microbiol..

[4] Cillidag S.I., Arcas N., Arroyo López F.N., Caballero J., D’Andria R., Fernández M., Fernandez E.R., Garrido A., López-Miranda J., Msallem M., Parras M. (2013). Table Olive Processing Technologies. Present and Future of the Mediterranean Olive Sector.

[5] Perpetuini G., Prete R., Garcia-Gonzalez N., Khairul Alam M., Corsetti A. (2020). Table Olives More than a Fermented Food. Foods.

[6] Bonatsou S., Tassou C., Panagou E., Nychas G.J. (2017). Table Olive Fermentation Using Starter Cultures with Multifunctional Potential. Microorganisms.

[7] Corsetti A., Perpetuini G., Schirone M., Tofalo R., Suzzi G. (2012). Application of Starter Cultures to Table Olive Fermentation: An Overview on the Experimental Studies. Front. Microbiol..

[8] Heperkan D. (2013). Microbiota of Table Olive Fermentations and Criteria of Selection for Their Use as Starters. Front. Microbiol..

[9] Argyri K., Doulgeraki A.I., Manthou E., Grounta A., Argyri A.A., Nychas G.J., Tassou C.C. (2020). Microbial Diversity of Fermented Greek Table Olives of Halkidiki and Konservolia Varieties from Different Regions as Revealed by Metagenomic Analysis. Microorganisms.

[10] Tzamourani A.P., Di Napoli E., Paramithiotis S., Oikonomou-Petrovits G., Panagiotidis S., Panagou E.Z. (2021). Microbiological and Physicochemical Characterization of Green Table Olives of Halkidiki and Conservolea Varieties Processed by the Spanish Method on Industrial Scale. Int. J. Food Sci. Technol..

[11] Panagou E.Z., Katsaboxakis C.Z. (2006). Effect of Different Brining Treatments on the Fermentation of Cv. Conservolea Green Olives Processed by the Spanish-Method. Food Microbiol..

[12] Lanza B. (2013). Abnormal Fermentations in Table-Olive Processing: Microbial Origin and Sensory Evaluation. Front. Microbiol..

[13] Bleve G., Tufariello M., Durante M., Grieco F., Ramires F.A., Mita G., Tasioula-Margari M., Logrieco A.F. (2015). Physico-Chemical Characterization of Natural Fermentation Process of Conservolea and Kalamàta Table Olives and Developement of a Protocol for the Pre-Selection of Fermentation Starters. Food Microbiol..

[14] Tsapatsaris S., Kotzekidou P. (2004). Application of Central Composite Design and Response Surface Methodology to the Fermentation of Olive Juice by *Lactobacillus plantarum* and *Debaryomyces hansenii*. Int. J. Food Microbiol..

[15] Ettayebi K., Errachidi F., Jamai L., Tahri-Jouti M.A., Sendide K., Ettayebi M. (2003). Biodegradation of Polyphenols with Immobilized Candida Tropicalis under Metabolic Induction. FEMS Microbiol. Lett..

[16] Hernández A., Martín A., Aranda E., Pérez-Nevado F., Córdoba M.G. (2007). Identification and Characterization of Yeast Isolated from the Elaboration of Seasoned Green Table Olives. Food Microbiol..

[17] Mantzouridou F., Tsimidou M.Z. (2011). Microbiological Quality and Biophenol Content of Hot Air-Dried Thassos Cv. Table Olives upon Storage. Eur. J. Lipid Sci. Technol..

[18] Roubos K., Moustakas M., Aravanopoulos F. (2010). Molecular Identification of Greek Olive (Olea Europaea) Cultivars Based on Microsatellite Loci. Genet. Mol. Res..

[19] International Olive Oil Council Table Olives Production. https://www.internationaloliveoil.org/wp-content/uploads/2020/12/OT-CE-901-23-11-2020-P.pdf.

[20] DOEPEL The Table Olive Sector in Numbers. https://doepel.gr/?page_id=15483&lang=en_US.

[21] Zoidou E., Melliou E., Gikas E., Tsarbopoulos A., Magiatis P., Skaltsounis A.L. (2010). Identification of Throuba Thassos, a Traditional Greek Table Olive Variety, as a Nutritional Rich Source of Oleuropein. J. Agric. Food Chem..

[22] Vekiari S.A., Oreopoulou V., Kourkoutas Y., Kamoun N., Msallem M., Psimouli V., Arapoglou D. (2010). Characterization and Seasonal Variation of the Quality of Virgin Olive Oil of the Throumbolia and Koroneiki Varieties from Southern Greece. Grasa Aceites.

[23] Kalogereas S.A. (1932). Table Olives.

[24] Panagou E.Z. (2006). Greek Dry-Salted Olives: Monitoring the Dry-Salting Process and Subsequent Physico-Chemical and Microbiological Profile during Storage under Different Packing Conditions at 4 and 20 °C. LWT—Food Sci. Technol..

[25] Koudounas K., Banilas G., Michaelidis C., Demoliou C., Rigas S., Hatzopoulos P. (2015). A Defence-Related Olea Europaea β-Glucosidase Hydrolyses and Activates Oleuropein into a Potent Protein Cross-Linking Agent. J. Exp. Bot..

[26] Hugenholtz P., Goebel B.M., Pace N.R. (1998). Impact of Culture-Independent Studies on the Emerging Phylogenetic View of Bacterial Diversity. J. Bacteriol..

[27] Ercolini D. (2013). High-Throughput Sequencing and Metagenomics: Moving Forward in the Culture-Independent Analysis of Food Microbial Ecology. Appl. Environ. Microbiol..

[28] Panagou E.Z., Schillinger U., Franz C.M.A.P., Nychas G.J. (2008). Microbiological and Biochemical Profile of Cv. Conservolea Naturally Black Olives during Controlled Fermentation with Selected Strains of Lactic Acid Bacteria. Food Microbiol..

[29] Bonatsou S., Paramithiotis S., Panagou E.Z. (2018). Evolution of Yeast Consortia during the Fermentation of Kalamata Natural Black Olives upon Two Initial Acidification Treatments. Front. Microbiol..

[30] Kazou M., Tzamourani A., Panagou E.Z., Tsakalidou E. (2020). Unraveling the Microbiota of Natural Black Cv. Kalamata Fermented Olives through 16S and ITS Metataxonomic Analysis. Microorganisms.

[31] Doulgeraki A.I., Hondrodimou O., Iliopoulos V., Panagou E.Z. (2012). Lactic Acid Bacteria and Yeast Heterogeneity during Aerobic and Modified Atmosphere Packaging Storage of Natural Black Conservolea Olives in Polyethylene Pouches. Food Control.

[32] Michailidou S., Trikka F., Pasentsis K., Petrovits G.E., Kyritsi M., Argiriou A. (2021). Insights into the Evolution of Greek Style Table Olives Microbiome Stored under Modified Atmosphere: Biochemical Implications on the Product Quality. Food Control.

[33] Visioli F., Galli C. (1998). Olive Oil Phenols and Their Potential Effects on Human Health. J. Agric. Food Chem..

[34] Kouka P., Priftis A., Stagos D., Angelis A., Stathopoulos P., Xinos N., Skaltsounis A., Mamoulakis C., Tsatsakis A., Spandidos D. (2017). Assessment of the Antioxidant Activity of an Olive Oil Total Polyphenolic Fraction and Hydroxytyrosol from a Greek Olea Europaea Variety in Endothelial Cells and Myoblasts. Int. J. Mol. Med..

[35] Pelucchi C., Bosetti C., Negri E., Lipworth L., La Vecchia C. (2011). Olive Oil and Cancer Risk: An Update of Epidemiological Findings through 2010. Curr. Pharm. Des..

[36] Soler-Rivas C., Espiń J.C., Wichers H.J. (2000). Oleuropein and Related Compounds. J. Sci. Food Agric..

[37] Alagna F., Mariotti R., Panara F., Caporali S., Urban S., Veneziani G., Esposto S., Taticchi A., Rosati A., Rao R. (2012). Olive Phenolic Compounds: Metabolic and Transcriptional Profiling during Fruit Development. BMC Plant Biol..

[38] Charoenprasert S., Mitchell A. (2012). Factors Influencing Phenolic Compounds in Table Olives (*Olea europaea*). J. Agric. Food Chem..

[39] Pang K.L., Chin K.Y. (2018). The Biological Activities of Oleocanthal from a Molecular Perspective. Nutrients.

[40] Lombardo G.E., Lepore S.M., Morittu V.M., Arcidiacono B., Colica C., Procopio A., Maggisano V., Bulotta S., Costa N., Mignogna C. (2018). Effects of Oleacein on High-Fat Diet-Dependent Steatosis, Weight Gain, and Insulin Resistance in Mice. Front. Endocrinol..

[41] Michailidou S., Petrovits G.E., Kyritsi M., Argiriou A. (2021). Amplicon Metabarcoding Data of Prokaryotes and Eukaryotes Present in ‘Kalamata’ Table Olives Packaged under Modified Atmosphere. Data Brief.

[42] Klindworth A., Pruesse E., Schweer T., Peplies J., Quast C., Horn M., Glöckner F.O. (2013). Evaluation of General 16S Ribosomal RNA Gene PCR Primers for Classical and Next-Generation Sequencing-Based Diversity Studies. Nucleic Acids Res..

[43] Chemidlin Prévost-Bouré N., Christen R., Dequiedt S., Mougel C., Lelièvre M., Jolivet C., Shahbazkia H.R., Guillou L., Arrouays D., Ranjard L. (2011). Validation and Application of a PCR Primer Set to Quantify Fungal Communities in the Soil Environment by Real-Time Quantitative PCR. PLoS ONE.

[44] Bolyen E., Rideout J.R., Dillon M.R., Bokulich N.A., Abnet C.C., Al-Ghalith G.A., Alexander H., Alm E.J., Arumugam M., Asnicar F. (2019). Reproducible, Interactive, Scalable and Extensible Microbiome Data Science Using QIIME 2. Nat. Biotechnol..

[45] Martin M. (2011). Cutadapt Removes Adapter Sequences from High-Throughput Sequencing Reads. EMBnet. J..

[46] Callahan B.J., McMurdie P.J., Rosen M.J., Han A.W., Johnson A.J.A., Holmes S.P. (2016). DADA2: High-Resolution Sample Inference from Illumina Amplicon Data. Nat. Methods.

[47] Quast C., Pruesse E., Yilmaz P., Gerken J., Schweer T., Yarza P., Peplies J., Glöckner F.O. (2013). The SILVA Ribosomal RNA Gene Database Project: Improved Data Processing and Web-Based Tools. Nucleic Acids Res..

[48] RStudio Team (2020). RStudio: Integrated Development for R. Rstudio.

[49] McMurdie P.J., Holmes S. (2012). Phyloseq: A Bioconductor Package for Handling and Analysis of High-Throughput Phylogenetic Sequence Data. Pac. Symp. Biocomput..

[50] Albertsen M., Karst S.M., Ziegler A.S., Kirkegaard R.H., Nielsen P.H. (2015). Back to Basics—The Influence of DNA Extraction and Primer Choice on Phylogenetic Analysis of Activated Sludge Communities. PLoS ONE.

[51] Wickham H., Gentleman R., Hornik K., Parmigiani G. (2016). Ggplot2.

[52] Boskou G., Salta F.N., Chrysostomou S., Mylona A., Chiou A., Andrikopoulos N.K. (2006). Antioxidant Capacity and Phenolic Profile of Table Olives from the Greek Market. Food Chem..

[53] Lanza B., Di Serio M.G., Iannucci E., Russi F., Marfisi P. (2010). Nutritional, Textural and Sensorial Characterisation of Italian Table Olives (*Olea europaea* L. Cv. ‘Intosso d’Abruzzo’). Int. J. Food Sci. Technol..

[54] Brand-Williams W., Cuvelier M.E., Berset C. (1995). Use of a Free Radical Method to Evaluate Antioxidant Activity. LWT—Food Sci. Technol..

[55] Capuzzo C., Firrao G., Mazzon L., Squartini A., Girolami V. (2005). “Candidatus Erwinia Dacicola”, a Coevolved Symbiotic Bacterium of the Olive Fly Bactrocera Oleae (Gmelin). Int. J. Syst. Evol. Microbiol..

[56] Bintsis T. (2017). Foodborne Pathogens. AIMS Microbiol..

[57] Arroyo-López F.N., Medina E., Ruiz-Bellido M.Á., Romero-Gil V., Montes-Borrego M., Landa B.B. (2016). Enhancement of the Knowledge on Fungal Communities in Directly Brined Aloreña de Málaga Green Olive Fermentations by Metabarcoding Analysis. PLoS ONE.

[58] Doulgeraki A.I., Pramateftaki P., Argyri A.A., Nychas G.J., Tassou C.C., Panagou E.Z. (2013). Molecular Characterization of Lactic Acid Bacteria Isolated from Industrially Fermented Greek Table Olives. LWT—Food Sci. Technol..

[59] Tzamourani A., Oikonomou-Petrovits G., Panagiotidis S., Nychas G.J., Panagou E. (2020). Effect of Packaging on Microbial Survival and Physicochemical Characteristics of Non-Thermally Preserved Green Spanish-Style Olives. Proceedings.

[60] Lucena-Padrós H., Caballero-Guerrero B., Maldonado-Barragán A., Ruiz-Barba J.L. (2014). Microbial Diversity and Dynamics of Spanish-Style Green Table-Olive Fermentations in Large Manufacturing Companies through Culture-Dependent Techniques. Food Microbiol..

[61] Kavroulakis N., Ntougias S. (2011). Bacterial and β-Proteobacterial Diversity in Olea Europaea Var. Mastoidis- and O. Europaea Var. Koroneiki-Generated Olive Mill Wastewaters: Influence of Cultivation and Harvesting Practice on Bacterial Community Structure. World J. Microbiol. Biotechnol..

[62] Campos C., Gomes L., Rei F.T., Nobre T. (2022). Olive Fruit Fly Symbiont Population: Impact of Metamorphosis. Front. Microbiol..

[63] Prohic A., Jovovic Sadikovic T., Krupalija-Fazlic M., Kuskunovic-Vlahovljak S. (2016). Malassezia Species in Healthy Skin and in Dermatological Conditions. Int. J. Dermatol..

[64] Costa D., Fernandes T., Martins F., Pereira J.A., Tavares R.M., Santos P.M., Baptista P., Lino-Neto T. (2021). Illuminating *Olea europaea* L. Endophyte Fungal Community. Microbiol. Res..

[65] Benítez-Cabello A., Ramiro-García J., Romero-Gil V., Medina E., Arroyo-López F.N. (2022). Fungal Biodiversity in Commercial Table Olive Packages. Food Microbiol..

[66] Benítez-Cabello A., Romero-Gil V., Medina-Pradas E., Garrido-Fernández A., Arroyo-López F.N. (2020). Exploring Bacteria Diversity in Commercialized Table Olive Biofilms by Metataxonomic and Compositional Data Analysis. Sci. Rep..

[67] Arroyo-López F.N., Romero-Gil V., Bautista-Gallego J., Rodríguez-Gómez F., Jiménez-Díaz R., García-García P., Querol A., Garrido-Fernández A. (2012). Yeasts in Table Olive Processing: Desirable or Spoilage Microorganisms?. Int. J. Food Microbiol..

[68] Hajjaj H., Blanc P., Groussac E., Uribelarrea J.L., Goma G., Loubiere P. (2000). Kinetic Analysis of Red Pigment and Citrinin Production by Monascus Ruber as a Function of Organic Acid Accumulation. Enzyme Microb. Technol..

[69] Panagou E.Z., Katsaboxakis C.Z., Nychas G.J. (2002). Heat Resistance of Monascus Ruber Ascospores Isolated from Thermally Processed Green Olives of the Conservolea Variety. Int. J. Food Microbiol..

[70] Panagou E.Z., Skandamis P.N., Nychas G.J. (2003). Modelling the Combined Effect of Temperature, PH and Aw on the Growth Rate of Monascus Ruber, a Heat-Resistant Fungus Isolated from Green Table Olives. J. Appl. Microbiol..

[71] Ramírez E., García-García P., De Castro A., Romero C., Brenes M. (2013). Debittering of Black Dry-Salted Olives. Eur. J. Lipid Sci. Technol..

[72] Sozbilen G.S., Baysal A.H. (2016). Microbial Profile and Bacterial Characterisation of Naturally Debittered Hurma Olives Compared to Non-debittered Erkence Variety during Ripening Period. Int. J. Food Sci. Technol..

[73] Panagou E.Z., Tassou C.C., Katsaboxakis K.Z. (2002). Microbiological, Physicochemical and Organoleptic Changes in Dry-salted Olives of Thassos Variety Stored under Different Modified Atmospheres at 4 and 20 °C. Int. J. Food Sci. Technol..

[74] Patriarca A. (2016). Alternaria in Food Products. Curr. Opin. Food Sci..

[75] Visconti A., Logrieco A., Bottalico A. (1986). Natural Occurrence of Alternaria Mycotoxins in Olives—Their Production and Possible Transfer into the Oil. Food Addit. Contam..

[76] Woods D.F., Kozak I.M., O’Gara F. (2020). Microbiome and Functional Analysis of a Traditional Food Process: Isolation of a Novel Species (Vibrio Hibernica) with Industrial Potential. Front. Microbiol..

[77] Breidt F., Medina E., Wafa D., Pérez-Díaz I., Franco W., Huang H.Y., Johanningsmeier S.D., Kim J.H. (2013). Characterization of Cucumber Fermentation Spoilage Bacteria by Enrichment Culture and 16S RDNA Cloning. J. Food Sci..

[78] Raimondi S., Luciani R., Sirangelo T.M., Amaretti A., Leonardi A., Ulrici A., Foca G., D’Auria G., Moya A., Zuliani V. (2019). Microbiota of Sliced Cooked Ham Packaged in Modified Atmosphere throughout the Shelf Life: Microbiota of Sliced Cooked Ham in MAP. Int. J. Food Microbiol..

[79] Tao Z., Wu X., Liu W., Takahashi H., Xie S., Ohshima C., He Q. (2022). Prevalence of Histamine-Forming Bacteria in Two Kinds of Salted Fish at Town Markets of Guangdong Province of South China. J. Food Prot..

[80] Illikoud N., Rossero A., Chauvet R., Courcoux P., Pilet M.F., Charrier T., Jaffrès E., Zagorec M. (2019). Genotypic and Phenotypic Characterization of the Food Spoilage Bacterium Brochothrix Thermosphacta. Food Microbiol..

[81] Ferrocino I., Rantsiou K., Cocolin L. (2022). Microbiome and -Omics Application in Food Industry. Int. J. Food Microbiol..

[82] Blekas G., Vassilakis C., Harizanis C., Tsimidou M., Boskou D.G. (2002). Biophenols in Table Olives. J. Agric. Food Chem..

[83] Marsilio V., Seghetti L., Iannucci E., Russi F., Lanza B., Felicioni M. (2005). Use of a Lactic Acid Bacteria Starter Culture during Green Olive (*Olea europaea* L. Cv Ascolana Tenera) Processing. J. Sci. Food Agric..

[84] Johnson R., Melliou E., Zweigenbaum J., Mitchell A.E. (2018). Quantitation of Oleuropein and Related Phenolics in Cured Spanish-Style Green, California-Style Black Ripe, and Greek-Style Natural Fermentation Olives. J. Agric. Food Chem..

[85] Mougiou N., Trikka F., Trantas E., Ververidis F., Makris A., Argiriou A., Vlachonasios K.E. (2018). Expression of Hydroxytyrosol and Oleuropein Biosynthetic Genes Are Correlated with Metabolite Accumulation during Fruit Development in Olive, Olea Europaea, Cv. Koroneiki. Plant Physiol. Biochem..

[86] Johnson R.L., Mitchell A.E. (2018). Reducing Phenolics Related to Bitterness in Table Olives. J. Food Qual..

[87] García-Serrano P., Brenes-Álvarez M., Romero C., Medina E., García-García P., Brenes M. (2023). Physicochemical and Microbiological Assessment of Commercial Dehydrated Black Olives. Food Control.

[88] López-García E., Benítez-Cabello A., Rodríguez-Gómez F., Romero-Gil V., Garrido-Fernández A., Jiménez-Díaz R., Arroyo-López F.N. (2022). Bacterial Metataxonomic Analysis of Industrial Spanish-Style Green Table Olive Fermentations. Food Control.

