# Supplementary figures and images for: Microbial and Biochemical Profile of Different Types of Greek Table Olives
Source: Foods. 2023 Apr 4;12(7):1527. doi: 10.3390/foods12071527 (PMC10094447; doi:10.3390/foods12071527)

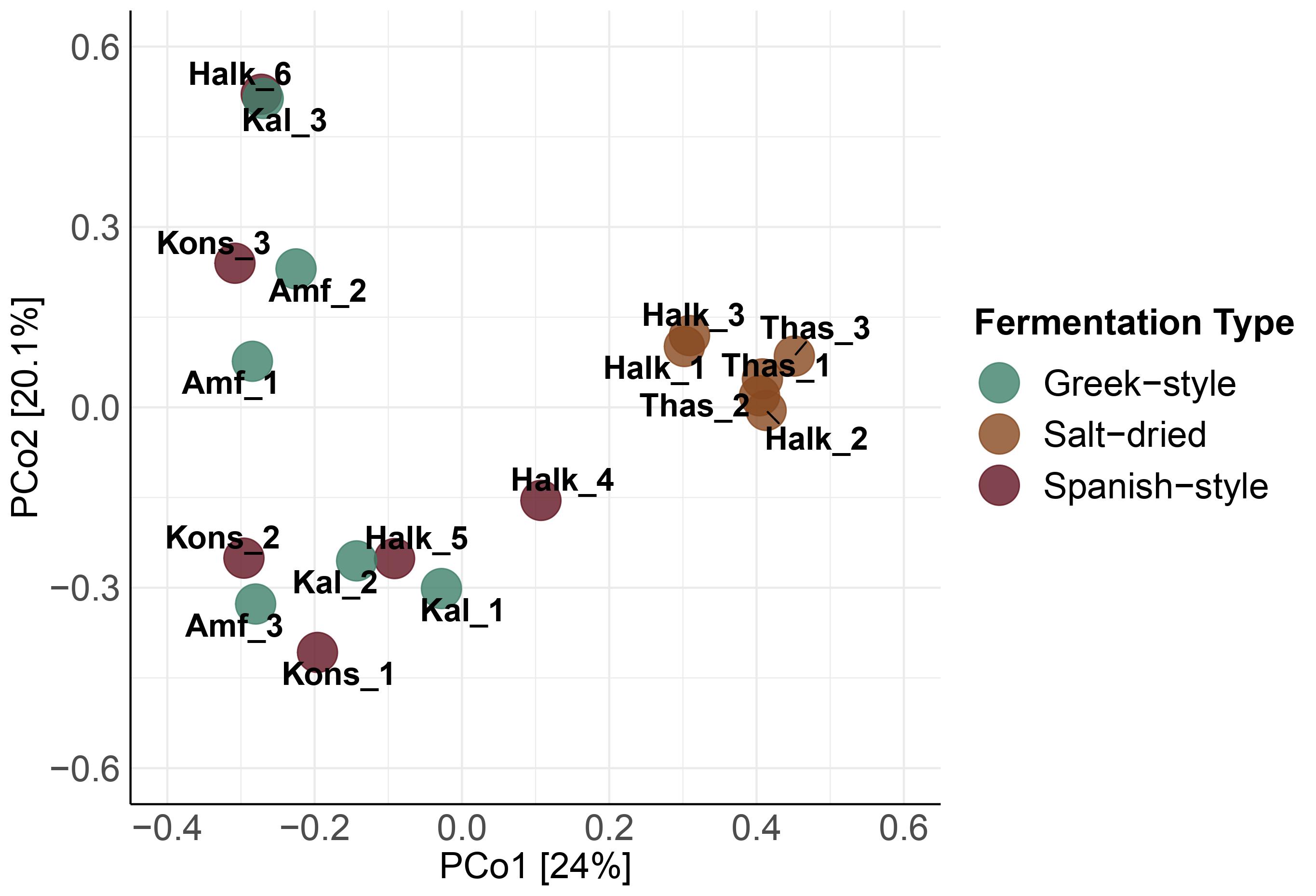

Supplement: Supplementary file 1 [file foods-12-01527-s001.zip › Supplementary Figure S1.jpg]

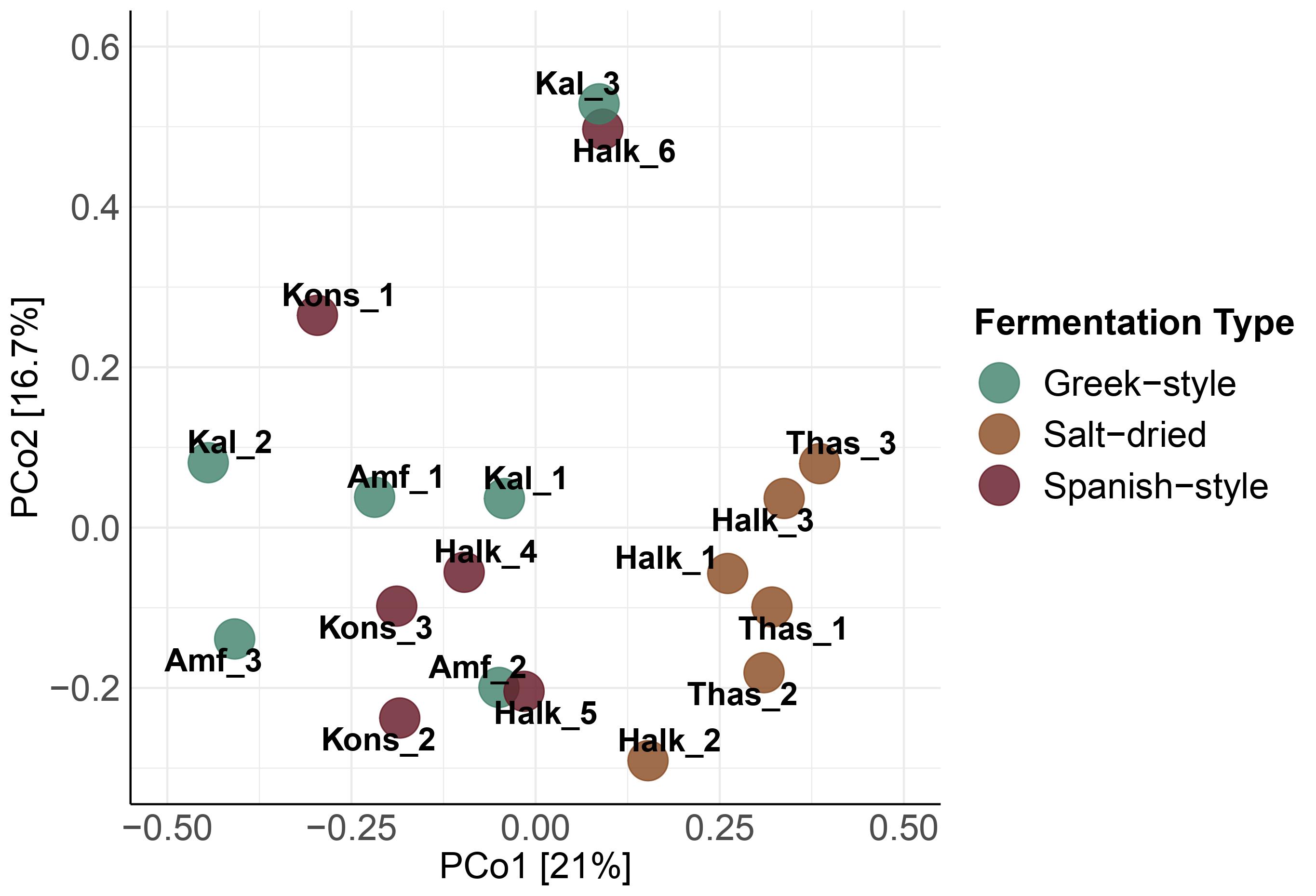

Supplement: Supplementary file 1 [file foods-12-01527-s001.zip › Supplementary Figure S2.jpg]
